# The Association Between Reading the Mind in the Eyes Test Performance and Intelligence Quotient in Children and Adolescents With Asperger Syndrome

**DOI:** 10.3389/fpsyt.2021.642799

**Published:** 2021-03-29

**Authors:** Inmaculada Peñuelas-Calvo, Aditya Sareen, Alejandro Porras-Segovia, Fanny-Beatriz Cegla-Schvatzman, Pablo Fernandez-Berrocal

**Affiliations:** ^1^Departamento de Psiquiatría Infantojuvenil, Hospital Universitario Fundación Jiménez Díaz, Madrid, Spain; ^2^Facultad de Psicología, Universidad Complutense de Madrid, Madrid, Spain; ^3^Instituto de Investigación Sanitaria Fundación Jiménez Díaz, Madrid, Spain; ^4^Bronxcare Health System, New York, NY, United States; ^5^Departamento de Psiquiatría, Hospital Universitario Fundación Jiménez Díaz, Madrid, Spain; ^6^Departamento de Psicología, Universidad de Málaga, Málaga, Spain

**Keywords:** RMET, intelligence, social cognition, autism spectrum disorder, reading the mind in eyes test, asperger syndrome

## Abstract

**Background:** There has been an extensive debate about a potential association between intelligence and social cognition. In this study, we aimed to assess the association between social cognition as measured with the Reading the Mind in the Eyes test (RMET) and intelligence as measured with the fourth edition of the Wechsler Intelligence Scale for Children (WISC-IV) in children and adolescents diagnosed with Asperger Syndrome (AS).

**Methods:** We conducted a cross-sectional study among 84 children diagnosed with AS aged 6–16 years (mean = 11.64; standard deviation = 2.75; 92.9% males). We analyzed the association between RMET performance and WISC-IV total score as well as the association between RMET performance and each of the four WISC-IV indexes (processing speed index, PSI; working memory index, WMI; perceptual reasoning index, PRI, and verbal comprehension index, VCI).

**Results:** We found a positive correlation between RMET performance and full-scale intelligence quotient (*r* = 0.340; *p* < 0.01), VCI (*r* = 0.310; *p* < 0.01), PRI (*r* = 0.401; *p* < 0.01), and WMI (*r* = 0.292; *p* < 0.01). In the linear regression model, age was a significant predictor of RMET score (β = 0.409; *p* < 0.001) as was PRI (β = 0.309; *p* = 0.019).

**Conclusion:** Our results suggest that intelligence quotient positively influences RMET performance, indicating that intelligence increases social cognition in individuals diagnosed with AS. However, weak-to-moderate size effects were found. This study contributes to understanding the mechanisms underlying the disturbance of social cognition in children and adolescents diagnosed with AS.

## Introduction

The theory of mind (ToM) is defined as the capacity to understand and empathize with others. It involves correctly attributing beliefs, desires, intentions, and perspectives to others and understanding that they are different from one's own (1). ToM is crucial to understand and predict others' behaviors, facilitating successful adaptation in different environments (2). The Reading the Mind in the Eyes test (RMET) was conceived as a ToM assessment tool, and it measures an essential aspect of social cognition: the ability to recognize emotional states in others by observing their eye expressions (3, 4). The RMET has shown good test–retest reliability in different cultural settings, including the Spanish population (5–8). The majority of children diagnosed with autism spectrum disorder (ASD) have impaired development of ToM (2, 9), which translates into lower performance on the RMET (3).

Intelligence has been widely studied among individuals diagnosed with ASD. Several cognitive scales are used in autism research to separate high- and low-functioning individuals and elucidate cognitive functioning in autism (10, 11). The Wechsler scales are the most widely used for measuring the intelligence quotient (IQ) in the ASD population (12). The Wechsler Intelligence Scale for Children–Fourth Edition (WISC-IV) is one of the most commonly used intelligence tests. It comprises four intelligence indexes, which represent different cognitive skills: verbal comprehension index (VCI), perceptual reasoning index (PRI), working memory index (WMI), and processing speed index (PSI). The WISC-IV score is expressed as the full-scale intelligence quotient (FSIQ), which combines the scores of the four WISC-IV indexes (13). The WISC-IV manual includes test scores results for special groups (14, 15) to provide information on the test's specificity and its clinical utility in the diagnostic evaluation. These special groups include individuals diagnosed with Asperger syndrome (AS), who usually obtain lower WMI and PSI scores, resulting in lower FSIQ (13, 16). On average, individuals diagnosed with AS show no significant language delay and no deficits in intelligence (13, 14). However, the difficulty in recognizing emotions is well-established in individuals with AS although it is not fully understood.

In children diagnosed with ASD, IQ may be associated with a better performance in social cognition tasks, including face-emotion recognition (17, 18). Intelligence may play a compensatory role in tasks that require emotional recognition (19). This compensatory role of intelligence could impact facial emotion recognition to a greater degree than in normal children (20).

Lately, the association between intelligence and RMET in ASD has gained some attention. In a recent meta-analysis, we explored the association between RMET performance and IQ in children diagnosed with ASD (21). We found that higher IQ was associated with higher RMET performance in controls, and in children with ASD, it had the opposite effect. However, results should be taken with caution as the meta-analysis included few studies.

In contrast, in the meta-analysis of Baker et al. (22), the authors found a positive correlation between IQ and RMET performance (*r* = 0.24) and concluded that both verbal and performance intelligence contributed equally to this association.

To our knowledge, no studies have assessed the association between different WISC-IV indexes and RMET performance in children diagnosed with AS. If certain indexes have a more significant impact than others in RMT performance, cognitive training programs could be tailored to tackle these deficits, thus improving social cognition in children diagnosed with AS. Cognitive training programs have been tested in children with ASD with good results (23) and (24). In turn, improvement in social cognition has been associated with greater functionality and quality of life (25).

We aimed to investigate whether RMET is associated with IQ as measured with the WISC-IV. We also aimed to identify which WISC-IV indexes—VCI, PRI, WMI, and PSI—were most strongly associated with RMET performance.

## Methods

This study was approved by the Regional Hospital Ethics Committee of Malaga. All procedures were conducted following the institutional research committee's ethical standards and with the 1964 Declaration of Helsinki and its later amendments. Informed consent was obtained from all participants' parents.

### Sample

This is a cross-sectional study carried out among children diagnosed with AS. Participants were recruited via the Asperger Syndrome Association of Málaga (Asociación Malagueña de Síndrome de Asperger, AMSA). The AMSA is a non-governmental, free-to-join association that does not have a direct relationship with clinical facilities. This association offers memberships to any person diagnosed with AS. AMSA members are representative of community-dwelling people with AS.

Inclusion criteria were:

1. Being aged between 6 and 16 years.
2. Having a diagnosis of AS by a clinical psychologist or psychiatrist as per the Diagnostic and Statistical Manual of Mental Disorders–Fourth Edition (DSM-IV) criteria (26) based on a clinical assessment using developmental history and a standardized scale, such as the Autism Diagnostic Interview–Revised (27) or the Autism Diagnostic Observation Schedule–Generic (28).
3. Having an IQ of 70 or higher on the WISC-IV.

There were no exclusion criteria regarding gender, ethnicity, or psychological/physical comorbidities.

### Measures

All assessments were carried out by trained clinicians. We used the Spanish version of the RMET for children (4, 29). This test includes 28 pictures of eye expressions. For each picture, participants must choose one of four possible corresponding emotional states (4). The children's version of the RMET has been validated in the Spanish population (29) with mean scores in typical children of 18.51 (*SD*: 3.93). This test is particularly recommended for children aged 8–17 although it has been employed in children as young as 6 with good results (30). In previous studies, children with high-functioning ASD scored lower than typical children (31).

We measured IQ with the Spanish version of the WISC-IV (13, 32). The WISC-IV was standardized for children aged 6–16. Norm tables were divided into 4-month age intervals. Children obtain scores that directly compare them with a representative sample of all children of the same age range (13). WISC-IV is composed of four indexes: VCI, PRI, WMI, and PSI. VCI measures verbal concept formation through the tasks similarities, vocabulary, and comprehension. PRI measures non-verbal and fluid reasoning with the tasks block design, picture concepts, and matrix reasoning subtests. WMI assesses children's working memory with the tasks digit span and letter–number sequencing. PSI measures processing speed with the tasks coding and symbol search.

### Procedure

If children and adolescents, as well as their parents, showed interest in the project, they were scheduled for a baseline interview in which the details of the project were explained fully, and they were given the informed consent form. They were given enough time to read the informed consent form, and any doubts they might have were answered. After signing the informed consent form, the standardized scales were administered by trained clinicians. Each participant was assessed individually using the RMET and the WISC-IV. Sessions lasted between 90 and 120 min. Participants were allowed to take short rest breaks (10–15 min) between tasks.

### Statistical Analysis

All analyses were carried out using the Statistical Package for the Social Sciences (SPSS), version 22.0. Preliminary analyses were conducted to compute descriptive statistics. The association between WISC-IV score and RMET performance was analyzed with zero-order Pearson's coefficients. Correlations between WISC-IV index scores and RMET performance were also conducted. According to Cohen (33), an absolute value of *r* ≤ 0.1 means weak correlation, an absolute value of 0.1–0.3 means moderate correlation, and an absolute value of ≥0.5 means large correlation. To explore the unique strengths of association between RMET score and WISC-IV indexes controlling by age, we conducted a two-step hierarchical linear regression analysis. In the first step, we introduced age, and in the second step, we introduced the four WISC-IV indexes. All tests were two-tailed with statistical significance at *p* < 0.05 and 95% confidence intervals.

## Results

### Description of the Sample

Our sample comprises 84 children diagnosed with AS with a mean age of 11.64 years (standard deviation = 2.75). The proportion of males was 92.9%. Table 1 shows the mean scores, standard deviations, and ranges for the WISC-IV scores, including FSIQ and the four index scores (VCI, PRI, WMI, PSI) as well as RMET scores.

**Table 1:** Demographics including age, WISC-IV index scores and FSIQ and RMET.

	**Mean**	**SD**	**Range**	
			**Min**	**Max**
Age (in years)	11.643	2.754	6	16
VCI	112.881	23.213	59	149
PRI	104.381	18.976	68	141
WMI	102.881	20.917	54	150
PSI	94.690	18.482	67	147
FSIQ	106.0	21.727	70	148
RMET	17.143	3.918	7	25

*FSIQ, Full Scale Intelligence Quotient; Min, Minimum; Max: Maximum; PRI, Perceptual Reasoning Index; PSI, Processing Speed Index; RMET, Reading the Mind in the Eyes Test; SD, Standard Deviation; WMI, Working Memory Index*.

### Correlation Results Between RMET Score and IQ

We found a weak positive correlation between RMET score and FSIQ (*r* = 0.215; *p* < 0.05), PRI (*r* = 0.287; *p* < 0.01), and WMI (*r* = 0.235; *p* < 0.05). We found a weak negative correlation between age and VCI (*r* = −0.221; *p* < 0.05), PRI (*r* = −0.237; *p* < 0.01), and FSIQ (*r* = −0.275; *p* < 0.05), a moderate negative correlation between age and PSI (*r* = −0.311; *p* < 0.01), and a moderate positive correlation between age and RMET score (*r* = 0.338; *p* < 0.05). Controlling by age, there was a moderate positive correlation between RMET score and VCI (*r* = 0.310; *p* < 0.01), PRI (*r* = 0.401; *p* < 0.01), and FSIQ (*r* = 0.340; *p* < 0.01) and a significant, weak positive between RMET score and WMI *(r* = 0.292; *p* < 0.01). No significant correlation was found between RMET score and PSI (*r* = 0.049; *p* > 0.10). Full results are shown in Table 2.

**Table 2:** Zero-order correlations among WISC-IV index scores and FSIQ.

	**VCI**	**PRI**	**WMI**	**PSI**	**FSIQ**	**RMET**	**Age**
VCI	1						
PRI	0.630^**^	1					
WMI	0.596^**^	0.516^**^	1				
PSI	0.358^**^	0.354^**^	0.407^**^	1			
*FSIQ*	0.870^**^	0.802^**^	0.786^**^	0.629^**^	1		
*RMET*	0.209	0.287^**^	0.235^*^	−0.061	0.215^*^	1	
*RMETpc*	0.310^**^	0.401^**^	0.292^**^	0.049	0.340^**^	1	
*Age*	−0.221^*^	−0.237^**^	−0.111	−0.311^**^	−0.275^*^	0.338^*^	1

*RMET and age and partial correlation controlling for age between WISC-IV index and RMET*.

** *p < 0.01*;

* *p < 0.05*.

*FSIQ, Full Scale Intelligence Quotient; PRI, Perceptual Reasoning Index; PSI, Processing Speed Index; RMET, Reading the Mind in the Eyes Test; RMETpc, Reading the Mind in the Eyes Test Partial Correlations; VCI, Verbal Comprehension Index; WMI, Working Memory Index*.

### Results of the Linear Regression Model

We carried out a linear regression model to explore the association between RMET score and WISC-IV indexes scores controlling by age. Results are shown in Table 3. The full model was statistically significant [*F*_(5, 78)_ = 6.094, *p* < 0.001], with a total R2 of 0.235. Age was a significant predictor of RMET score (β = 0.409, *p* < 0.001). Thus, older children showed higher RMET scores than younger children. PRI was also significantly associated with RMET score (β = 0.309, *p* = 0.019), showing that children with higher PRI scored higher on the REMT.

**Table 3:** Hierarchical linear regression model: associations between RMET score and WISC-IV indexes controlled by age.

**Predictors**	**Beta**	**Adjusted *R*^**2**^ change**	***F* change**	***p***
Step 1: Age	0.339	0.115	10.626	0.002
Step 2: Age + WISC-IV indexes		0.166	4.507	0.003
Age	0.409			<0.001
PRI	0.309			0.019
PSI	−0.112			0.318
VCI	0.013			0.576
WMI	0.129			0.313

*PRI, Perceptual Reasoning Index; PSI, Processing Speed Index; RMET, Reading the Mind in the Eyes Test; VCI, Verbal Comprehension Index; WMI, Working Memory Index*.

*Snell R^2^ Total = 0.235, F_(5, 78)_ = 6.094, p < 0.001*.

**Figure 1:**
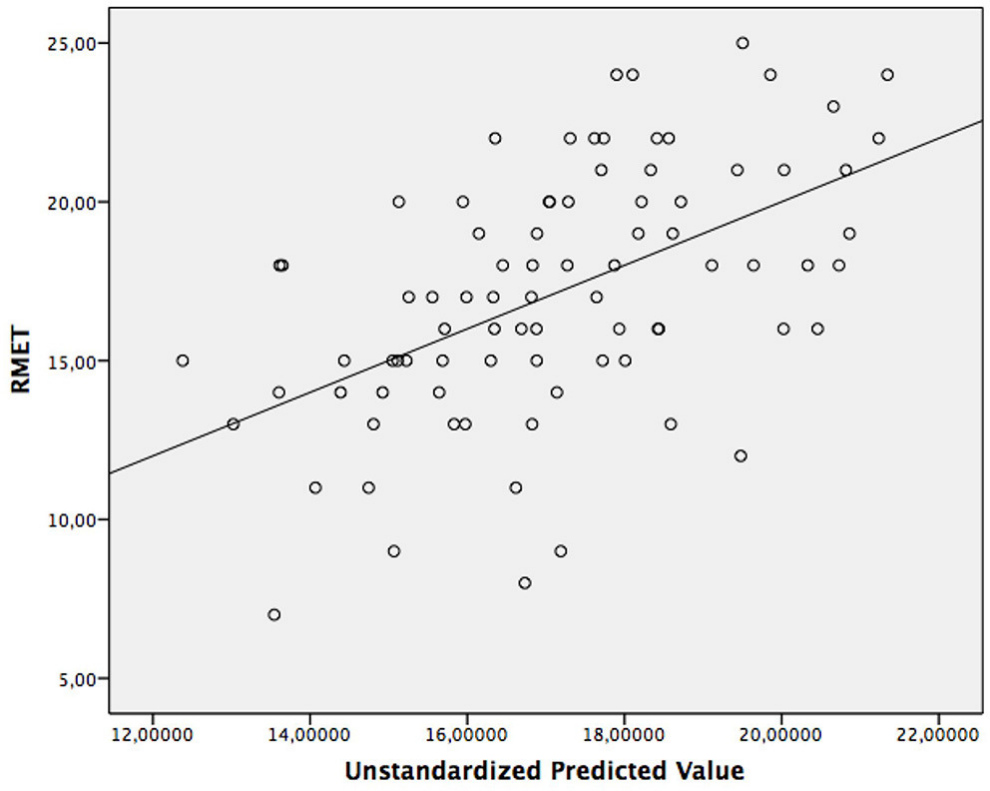
Scatterplot of RMET and unstandardized predicted values for the final regression model with a linear fit line.

Figure 1 shows a scatterplot of RMET scores and unstandardized predicted values for the linear regression model (Figure 1).

## Discussion

### Summary of Results

This study explored the association between RMET performance and IQ in children and adolescents with AS. After controlling by age, we found a moderate positive correlation between RMET performance and FSIQ, VCI, and PRI and a weak positive correlation between RMET and WMI. In the linear regression analysis, we found that age and PRI were statistically significantly associated with RMET performance. Thus, perceptual components of intelligence are the areas that appear more related to social cognition. Verbal component and working memory also appear to play a role as does total IQ. However, processing speed was not associated with RMET.

We found a moderate positive correlation between age and RMET, suggesting that age might be an important factor in determining RMET performance in children and adolescents. Future research should determine whether these findings might be confirmed in other age groups as well as in longitudinal studies.

### Comparison With Previous Research

The RMET mean score found in our sample (17.143, *SD*: 3.918) is slightly lower than the mean score found in the Spanish validation of the RMET, which was performed in a sample of typical children (mean score: 18.51, SD: 3.93) (29). Also, the distribution of WISC-IV index scores found in our sample was similar to that reported in the WISC-IV manual (lowest scores were WMI and PSI) (32). Some previous studies on individuals diagnosed with AS found no correlation between RMET performance and IQ (3, 4, 34, 35). In contrast, our study suggests that higher IQ attributes and higher RMET performance are associated.

Regarding visual-perceptual processing, we found that PRI is correlated with RMET performance. The PRI was, in fact, the only WISC-IV index significantly associated with RMET performance in the linear regression analysis. In contrast, some studies claim that difficulties in interpreting facial emotions are not traits of the visual-perceptual process (36, 37). However, patients diagnosed with AS frequently report abnormal sensory experiences (38). Also, in previous studies, individuals diagnosed with AS obtained better PRI scores than normal individuals (39, 40). As PRI refers to non-verbal intelligence, it requires accurate visual processing and then reasoning. Therefore, individuals diagnosed with AS with a better reasoning ability may perform better in RMET.

Similarly, we found a moderate positive correlation between VCI and RMET performance. Studies show that people diagnosed with ASD have pragmatic language problems (41), especially those with AS (42). This deficit may be related to weak central coherence, which is the difficulty in adjusting language to context as (43) described.

WMI was also associated with RMET performance in our study. Working memory (WM) is an essential part of executive functioning. Previous studies have found that individuals diagnosed with AS have deficits in WM after controlling for the influence of IQ (44). Finally, we found no correlation between PSI and RMET performance.

### Mechanisms Underlying the Association

Different mechanisms could explain the association between intelligence and social cognition. On the one hand, social cognition and intelligence could share functional brain areas. Thus, the improvement in one area may influence the improvement in the other area and vice versa. For instance, a study showed that social cognition training increased cortical thickness in prefrontal regions (45). Conversely, cognitive training is shown to improve facial affect recognition (23).

On the other hand, higher intelligence generally increases the ability to solve problems, so individuals with AS could use their intelligence as a compensatory strategy to recognize facial expressions and other emotional indicators in the people around them. Deficits in the so-called emotional brain, which involves areas such as the amygdala, could explain the social atypicalities found in people with ASD (46). In this regard, a study used functional magnetic resonance imaging to explore which areas were activated in people with ASD and healthy controls. During an eye expression recognition task, two areas of the so-called social brain—the amygdala and the superior temporal gyrus—and the prefrontal cortex were activated in healthy controls. However, individuals with ASD did not activate their amygdala during the task, but instead relied on the activation of frontotemporal regions to perform the task (20).

### Limitations

Our findings should be considered in light of some limitations. First, the cross-sectional design of our study precludes drawing conclusions about the causality of the associations found. Likewise, we cannot establish the directionality of these associations. Therefore, we should also consider the possibility that social cognition leads to better cognitive development and a higher IQ. Also, although all participants had a total IQ of 70 or above, some of them scored below 70 in some of the indexes. Finally, most of the sample was composed of males, so the conclusions derived from our study are difficult to extrapolate to the female population.

## Conclusions

In conclusion, our results suggest an association between RMET performance and IQ in children diagnosed with AS. Particularly, the perceptual reasoning component was the most strongly associated with RMET. This association should be considered when evaluating RMET performance. Our results suggest that intelligence positively influences social cognition although causality cannot be established. Also, moderate size effects were found, which requires us to take these results with caution. Longitudinal research could be useful to clarify the association between social cognition and intelligence.

Among the mechanisms that could explain the association between intelligence and social cognition, the overlapping of brain areas involved in both functions and the use of intelligence as a compensatory strategy stand out. Cognitive training could be useful in improving social cognition in children and adolescents with AS. In turn, the improvement of social cognition could lead to greater functionality and quality of life in this population.

## Data Availability Statement

The original contributions generated for the study are included in the article/Supplementary Material, further inquiries can be directed to the corresponding authors.

## Ethics Statement

The studies involving human participants were reviewed and approved by Regional Hospital Ethics Committee of Malaga. Written informed consent to participate in this study was provided by the participants' legal guardian/next of kin.

## Author Contributions

IP-C designed the study, performed the data collection and scoring, and wrote the article. AS wrote the article and contributed to the final version. AP-S contributed to the search for references and contributed to the final version. F-BC-S contributed to the search for references and contributed to the final version. PF-B designed the study performed the statistical analyses and contributed to the final version.

## Conflict of Interest

The authors declare that the research was conducted in the absence of any commercial or financial relationships that could be construed as a potential conflict of interest.
