# Supplementary material for: The Association Between Reading the Mind in the Eyes Test Performance and Intelligence Quotient in Children and Adolescents With Asperger Syndrome
Source: Front Psychiatry. 2021 Mar 29;12:642799. doi: 10.3389/fpsyt.2021.642799 (PMC8039142; doi:10.3389/fpsyt.2021.642799)
Supplement: Supplementary file 1 [file Data_Sheet_1.PDF]

#Participant	Gender	AGE	Similarities	Vocabulary	Comprehension	Block Design	Picture conceps	Matrix Reasoning	Digit Span	Letter-Number	Coding	Symbol Search	VCI	PRI	WMI	PSI	FSIQ	MET total sco	RMET1	RMET2	RMET3	RMET4	RMET5	RMET6	RMET7	RMET8	RMET9	RMET10	RMET11	RMET12	RMET13	RMET14	RMET15	RMET16	RMET17	RMET18	RMET19	RMET20	RMET21	RMET22	RMET23	RMET24	RMET25	RMET26	RMET27	RMET28
1	M	9	19	19	6	10	18	18	15	13	6	9	126	132	122	88	132	21	1	4	2	2	2	4	3	1	4	3	3	4	3	2	1	1	4	1	4	3	1	3	2	1	1	3	3	4
2	M	9	19	19	17	12	16	15	17	16	11	17	147	127	137	121	145	22	4	1	4	2	2	3	3	1	4	3	2	4	2	2	4	1	1	1	4	3	1	4	2	1	4	3	3	3
3	M	8	10	4	3	6	11	9	8	3	4	6	75	91	75	73	73	18	4	4	4	2	2	4	3	1	4	3	2	4	2	2	1	4	4	1	2	4	1	4	2	2	4	4	3	4
4	M	10	9	10	9	2	9	4	4	3	5	3	97	69	55	77	70	13	3	3	1	1	2	4	4	1	4	3	2	4	4	4	1	2	4	3	4	3	2	1	4	2	1	3	2	1
5	M	10	12	11	11	13	9	6	8	10	9	12	108	95	93	104	100	15	1	4	4	2	2	1	3	1	3	2	2	4	3	4	1	1	1	1	1	3	1	3	2	3	2	3	3	1
6	M	9	19	15	18	14	13	12	15	14	6	7	142	118	125	82	125	21	4	4	2	2	2	4	3	3	4	3	2	4	3	2	1	1	4	1	4	3	1	4	2	1	2	3	3	4
7	M	9	14	9	11	13	7	12	10	8	11	11	108	103	93	107	107	14	4	4	4	3	2	4	3	1	3	3	4	4	1	3	1	1	3	1	2	3	1	4	2	2	2	4	2	1
8	M	10	3	11	9	8	10	7	10	5	13	7	89	91	85	102	86	15	4	1	4	2	2	4	3	1	2	3	2	1	1	3	2	1	4	1	2	3	2	4	2	1	4	4	2	2
9	M	9	16	13	11	17	10	13	16	6	13	11	120	120	105	112	120	17	4	2	2	2	2	3	3	1	3	3	2	4	2	2	1	3	4	1	4	3	1	3	1	1	1	4	3	1
10	M	10	18	17	16	10	11	11	14	13	8	10	142	103	120	93	122	19	4	4	2	2	2	4	4	1	1	3	4	4	1	2	3	1	4	1	4	3	1	4	2	1	2	3	3	4
11	M	10	19	18	19	10	13	10	15	5	6	9	149	105	99	88	119	8	4	3	2	2	2	3	2	1	3	2	4	1	2	4	1	1	1	1	2	2	1	2	1	4	3	2	4	4
12	M	9	10	7	7	6	7	6	10	7	7	6	89	77	91	82	78	14	2	3	2	3	1	3	3	1	3	3	3	2	1	2	1	4	1	1	4	3	1	3	1	1	4	3	1	4
13	M	10	16	15	15	12	13	13	15	15	13	11	131	116	127	112	128	20	4	4	1	2	2	3	2	1	4	1	2	4	1	4	1	1	4	1	3	3	1	3	2	1	2	4	3	3
14	M	11	19	18	16	5	15	13	15	13	7	7	122	85	105	143	122	14	3	4	3	2	4	3	3	4	4	2	2	4	3	4	3	1	4	1	2	3	1	3	1	3	2	4	3	2
15	M	11	16	12	11	16	16	15	15	15	6	6	118	134	122	79	120	17	1	4	4	2	2	3	3	1	4	3	2	4	1	2	4	1	1	1	2	1	2	4	2	1	1	3	4	1
16	M	10	15	12	10	14	15	14	12	11	6	5	113	127	110	73	109	16	1	4	4	2	1	2	2	1	1	3	2	4	1	2	1	4	4	1	3	3	1	4	2	1	2	4	1	2
17	M	11	19	11	10	16	17	12	9	10	4	5	120	131	97	70	109	18	1	1	1	2	2	4	3	1	4	1	2	4	1	2	1	4	2	1	4	3	2	2	2	1	4	1	2	3
18	M	9	11	12	9	11	15	11	15	11	11	18	105	114	116	124	124	16	4	4	4	2	2	4	3	4	3	3	2	4	2	2	1	1	3	1	2	3	3	4	2	1	2	4	3	1
19	M	11	15	18	16	11	6	17	9	15	13	15	136	107	110	121	126	16	4	4	4	2	1	3	3	4	4	3	1	3	1	4	1	1	1	1	4	3	1	1	2	3	4	1	3	1
20	M	10	19	18	19	11	15	12	13	11	4	9	149	116	110	82	124	18	1	4	4	1	2	4	3	1	4	3	1	4	1	4	1	1	3	1	4	3	1	4	2	1	1	4	3	4
21	M	7	16	19	14	16	17	11	9	3	14	12	136	129	75	117	114	15	1	1	2	2	2	4	3	4	4	2	4	4	1	2	1	1	1	1	4	3	1	3	2	3	1	2	3	1
22	M	7	19	9	15	16	15	15	19	17	10	17	144	119	132	125	139	15	4	4	4	2	1	2	3	1	4	1	2	4	2	2	1	4	4	2	4	3	1	4	3	1	1	4	1	2
23	F	7	13	9	7	10	14	14	11	9	16	15	99	116	99	130	113	11	1	1	3	1	2	4	3	1	2	3	2	1	2	4	1	1	3	3	2	2	1	3	2	1	2	1	4	3
24	M	7	19	19	13	13	15	10	15	10	10	11	139	116	114	104	130	17	4	4	2	2	1	4	3	1	4	1	2	4	1	2	1	3	4	1	4	2	3	4	2	1	4	2	1	1
25	M	7	19	17	19	16	17	19	19	15	10	13	139	110	144	147	148	11	3	4	4	4	4	4	3	1	4	2	1	4	2	1	4	1	3	2	1	3	4	4	1	1	1	4	3	1
26	M	8	18	19	16	14	8	13	19	19	9	11	143	109	150	102	135	11	3	1	1	4	4	4	4	1	3	3	2	4	1	1	1	1	4	4	2	4	2	3	2	2	3	4	2	1
27	M	13	8	6	4	7	9	7	6	4	5	7	77	86	71	78	73	17	4	3	2	4	2	3	3	1	3	3	3	4	3	2	3	1	3	1	4	3	1	4	2	1	2	3	3	4
28	M	11	17	15	18	12	11	8	13	11	8	11	137	101	110	99	119	16	1	4	4	1	2	4	3	1	4	3	2	4	4	2	3	1	4	1	4	3	3	2	2	3	3	4	3	1
29	M	10	15	7	9	11	8	4	13	4	3	5	103	85	91	67	82	17	2	4	1	1	2	4	3	1	4	2	4	4	3	2	3	2	4	1	2	3	1	4	2	1	4	4	3	1
30	F	12	15	11	8	12	16	11	11	12	5	9	108	118	108	85	107	22	1	4	2	2	2	3	3	1	4	3	2	4	1	2	1	1	4	3	2	3	1	4	2	1	4	4	3	4
31	M	12	14	11	14	10	12	13	12	13	6	6	114	79	109	118	107	15	3	4	1	2	4	4	2	1	4	2	3	4	1	4	1	1	3	1	4	4	3	1	2	1	1	1	4	3
32	M	13	14	11	13	17	15	17	15	13	7	10	116	141	122	93	124	19	1	4	4	2	2	4	3	1	4	3	2	4	1	2	1	1	4	4	4	1	3	4	2	1	3	1	4	3
33	M	13	15	19	16	17	12	14	9	11	8	11	137	127	99	99	124	12	2	1	4	1	3	4	3	1	4	1	4	4	2	2	3	1	3	1	2	3	1	1	2	1	2	4	2	3
34	M	14	13	6	4	6	14	5	9	5	7	8	87	90	83	85	83	20	1	4	1	2	2	3	2	1	4	2	2	4	2	2	1	1	4	2	4	3	1	4	2	1	4	2	1	4
35	M	14	15	13	9	7	9	8	7	10	11	12	99	85	75	97	84	16	2	4	4	1	4	3	3	1	4	1	4	4	4	3	4	1	4	4	4	3	1	4	2	1	2	3	3	4
36	M	13	17	15	16	14	14	19	16	13	6	10	134	134	125	91	130	17	1	4	2	2	2	2	3	1	1	3	1	4	1	4	4	1	3	3	4	3	1	4	2	1	4	3	3	4
37	M	14	11	12	5	11	11	11	13	11	7	13	97	105	110	102	105	21	1	4	4	2	2	4	3	4	4	3	2	4	2	2	1	1	4	1	4	3	1	4	2	1	2	3	3	4
38	F	15	12	11	10	4	10	7	12	15	4	6	107	81	120	73	91	16	1	4	4	2	2	2	4	1	4	3	2	4	2	2	1	1	4	2	4	2	1	4	2	3	1	2	2	4
39	M	14	12	13	11	6	7	9	12	11	8	8	113	83	108	91	97	22	4	4	4	2	2	4	3	1	4	3	3	4	1	2	1	1	1	1	4	3	1	4	2	1	4	3	3	4
40	M	13	19	17	17	4	14	12	19	13	13	13	143	100	133	117	131	24	1	4	1	2	2	4	3	1	4	1	2	4	1	2	1	1	4	1	4	3	1	4	2	1	4	1	3	3
41	M	13	18	19	12	17	13	19	15	11	5	8	136	141	116	82	128	22	4	4	4	1	2	3	3	1	1	1	2	4	1	2	1	1	4	1	4	3	3	4	2	1	4	3	3	3
42	M	12	17	16	14	15	15	19	15	16	11	15	132	141	130	117	140	21	4	1	4	2	2	3	3	1	4	3	2	4	1	1	1	1	1	1	4	3	1	4	2	1	4	4	4	3
43	M	16	7	4	3	6	5	4	9	9	4	8	70	71	93	79	70	14	2	4	1	2	4	3	3	1	2	1	1	4	4	4	4	3	2	1	4	2	2	4	1	1	4	3	2	3
44	M	16	16	17	17	10	15	4	4	9	4	6	137	97	79	73	101	21	4	1	1	2	2	3	3	1	4	3	2	4	1	2	4	1	4	1	4	1	1	4	2	1	4	2	4	4
45	M	15	12	12	11	11	8	16	8	12	6	8	110	109	99	85	102	25	1	4	4	2	2	4	3	1	4	3	2	4	1	2	1	1	4	1	4	3	1	4	2	1	4	3	3	3
46	M	10	12	13	10	10	12	9	12	11	11	14	108	103	108	115	110	14	4	4	4	1	2	4	4	1	4	3	1	4	1	4	1	1	3	1	2	3	4	4	2	1	2	4	1	4
47	M	14	13	13	6	9	3	12	3	10	4	8	85	87	79	79	78	15	4	1	1	2	2	4	3	1	4	3	4	1	2	2	1	1	4	4	4	3	1	4	1	3	1	4	2	4
48	M	16	16	16	12	14	15	16	12	15	18	17	126	131	120	138	139	21	3	2	1	2	4	4	3	1	4	3	2	4	1	2	4	1	4	4	4	3	1	4	2	1	4	2	2	3
49	M	11	19	19	10	14	16	15	7	15	11	12	134	131	105	110	128	22	3	4	4	2	2	3	3	1	4	3	2	4	1	3	1	1	4	3	4	3	2	4	2	1	4	4	2	3
50	M	7	12	7	6	8	6	12	13	11	9	5	91	91	110	91	91	17	3	2	1	2	1	4	3	1	1	4	2	4	1	4	1	1	3	1	4	3	4	4	2	1	2	3	3	4
51	M	8	12	7	4	5	9	8	8	5	4	5	89	84	80	70	76	7	2	4	4	2	1	4	4	2	4	1	4	1	2	1	4	3	2	4	4	2	4	4	2	1	1	4	4	1
52	M	8	17	15	10	11	14	10	9	11	3	7	123	109	99	73	104	20	3	2	3	2	2	3	2	1	4	3	4	4	1	2	1	1	1	1	2	3	2	4	2	1	3	3	3	3
53	M	8	13	13	17	10	11	12	13	8	13	10	125	105	102	110	115	13	4	4	2	4	2	3	2	4	4	3	4	4	2	2	1	4	3	3	4	3	3	2	3	3	4	1	3	3
54	F	6	14	5	3	6	11	9	11	4	8	8	85	91	85	91	82	14	4	4	3	2	2	3	4	1	3	1	4	2	1	4	2	1	4	4	4	3	3	4	2	1	4	3	4	1
55	M	8	19	16	12	14	13	16	10	11	6	11	132	127	102	91	121	18	4	4	1	2	4	4	3	1	4	2	2	4	1	4	4	1	4	1	4	3	3	4	3	1	4	4	3	4
56	M	15	19	17	14	13	12	12	14	12	9	8	137	114	116	93	123	18	1	4	4	2	2	4	3	1	4	3	2	4	4	3	1	1	4	1	2	3	1	1	2	2	2	3	3	4
57	M	11	14	11	8	8	9	9	15	6	4	5	107	91	102	70	90	18	1	4	4	2	2	4	3	1	4	2	1	4	1	3	1	1	3	3	1	3	1	4	2	1	4	3	3	4
58	M	12	18	15	18	16	19	14	13	17	10	19	139	141	127	124	145	23	1	4	1	2	2	3	3	1	4	3	1	4	1	2	1	1	4	1	4	3	1	4	2	1	4	4	3	4
59	M	12	17	16	14	14	8	13	19	18	10	12	132	109	147	107	131	13	2	1	4	2	2	4	4	4	1	3	2	4	2	2	4	3	4	1	2	3	3	4	2	1	2	4	3	1
60	M	13	15	11	15	9	10	11	11	8	3	9	122	100	97	79	101	15	4	1	4	2	1	4	1	1	4	2	1	4	1	2	1	1	1	2	2	3	1	4	2	1	3	3	4	3
61	M	14	19	19	18	12	13	12	12	14	6	9	149	114	116	88	126	16	1	1	2	1	2	4	3	1	2	3	4	4	2	2	4	1	4	3	4	3	1	4	2	1	2	3	3	4
62	M	11	12	19	8	10	11	8	9	14	11	9	118	97	108	99	107	22	1	4	4	2	2	2	3	1	1	3	2	4	1	2	1	1	4	1	1	3	1	4	2	1	2	3	3	3
63	M	10	19	15	11	12	11	15	13	11	9	10	128	116	110	99	120	9	2	4	4	2	3	4	3	4	1	2	4	4	4	2	3	1	4	4	2	3	4	1	2	2	2	4	4	1
64	M	11	5	2	2	13	9	11	2	3	9	9	59	107	54	99	75	9	1	4	1	1	4	3	1	4	4	2	2	4	1	2	1	2	3	3	2	2	3	2	3	2	2	4	4	2
65	M	16	9	7	5	3	11	9	9	11	4	5	83	87	99	70	79	19	3	4	4	2	4	3	3	1	1	3	1	4	1	4	1	1	3	1	4	3	1	4	2	1	3	3	3	1
66	M	14	10	7	6	7	6	3	7	6	4	6	87	75	79	72	73	15	4	1	3	2	2	4	3	1	1	2	2	4	1	2	3	1	3	1	2	3	1	4	2	2	1	1	3	1
67	M	16	19	13	12	8	14	10	14	12	5	4	126	103	116	70	107	23	4	4	1	2	2	3	2	1	4	3	2	4	1	2	1	1	1	1	4	3	1	4	2	1	2	4	3	3
68	M	15	14	12	9	12	11	9	10	10	10	8	116	100	99	99	104	20	1	3	1	1	2	3	3	1	1	3	2	4	1	4	1	1	4	1	2	3	1	1	2	1	2	4	3	3
69	M	15	5	5	3	1	7	5	17	13	6	5	70	74	127	76	78	17	2	4	4	2	1	3	4	1	4	3	2	4	1	4	4	1	4	3	3	3	3	4	2	2	4	2	3	3
70	M	13	7	8	8	13	15	7	6	8	9	9	87	104	82	97	91	20	1	4	1	2	1	4	3	1	2	3	2	4	2	4	1	1	4	1	2	3	1	4	2	2	4	3	3	3
71	M	16	15	17	16	14	11	13	16	13	6	9	134	116	125	88	123	24	3	4	2	2	2	3	3	1	4	3	2	4	1	2	3	1	4	1	4	3	1	4	2	1	2	3	3	4
72	F	16	13	15	10	10	12	13	13	15	5	10	114	107	122	88	110	16	4	4	1	2	1	3	2	1	4	3	2	1	1	3	2	1	1	1	2	3	3	1	2	1	1	2	3	3
73	M	15	9	8	12	1	7	6	2	3	7	6	99	85	54	82	77	16	4	2	1	2	2	3	3	1	2	1	1	4	4	2	4	4	1	1	4	3	1	4	2	1	3	3	4	3
74	M	11	8	9	9	4	10	10	9	8	9	10	93	88	91	97	88	19	3	1	1	2	2	3	3	1	4	3	2	4	2	4	4	1	4	1	4	3	1	1	2	1	2	1	2	3
75	M	15	10	9	9	2	9	3	4	2	5	6	97	68	54	76	70	13	1	2	4	2	2	3	1	4	4	3	4	4	2	2	4	1	4	4	4	1	3	1	2	1	3	2	3	1
76	M	15	3	12	6	12	9	4	15	9	12	9	83	90	110	103	92	23	1	4	4	2	2	4	3	1	4	3	2	4	2	2	1	1	3	1	4	3	1	4	2	1	4	4	3	3
77	M	13	9	13	6	6	8	6	8	8	6	8	97	79	88	85	82	12	2	4	4	2	2	4	3	1	1	3	4	4	1	4	3	1	4	1	2	3	3	1	2	2	1	4	4	1
78	M	14	12	7	1	7	9	12	9	1	5	5	82	95	68	73	76	18	1	2	2	2	2	3	3	4	4	3	4	1	1	2	4	1	4	1	2	3	1	4	2	3	4	3	3	1
79	M	13	10	13	5	5	6	8	7	5	7	9	85	83	80	76	80	19	1	4	4	2	2	4	3	4	4	3	2	4	1	2	1	1	4	4	4	3	1	4	2	1	2	4	4	4
80	M	14	8	7	4	9	12	8	11	9	9	6	80	97	99	88	85	15	1	4	4	2	2	4	4	4	4	3	2	1	2	3	4	1	4	1	1	3	1	4	2	1	1	4	4	3
81	F	15	7	1	4	6	8	7	8	11	8	10	68	81	97	97	84	13	3	1	4	2	2	4	2	1	1	3	3	4	4	1	4	1	2	2	4	3	2	4	3	1	4	2	3	2
82	M	11	15	7	6	17	15	13	10	13	11	12	97	129	108	110	113	16	4	4	2	2	2	3	4	1	1	1	1	4	1	4	1	1	4	4	4	3	3	1	2	1	1	3	1	3
83	M	12	19	15	18	14	9	16	7	9	11	11	142	118	88	107	122	24	3	4	2	2	2	4	3	1	4	3	2	4	1	2	4	1	4	1	4	3	1	4	2	1	4	3	3	1
84	M	13	10	11	14	13	8	13	9	10	7	7	110	107	97	85	92	17	1	4	4	2	2	4	3	1	4	3	2	4	3	2	4	1	4	1	1	3	1	4	2	3	2	3	3	4
